# Conformational and Functional Properties of the Bioactive Thiosemicarbazone and Thiocarbohydrazone Compounds

**DOI:** 10.3390/cimb47090676

**Published:** 2025-08-22

**Authors:** Nikitas Georgiou, Ektoras Vasileios Apostolou, Stamatia Vassiliou, Demeter Tzeli, Thomas Mavromoustakos

**Affiliations:** 1 Laboratory of Organic Chemistry, Department of Chemistry, National and Kapodistrian University of Athens, Panepistimioupolis Zografou, 15771 Athens, Greece; nikitage@chem.uoa.gr (N.G.); ektorasap@chem.uoa.gr (E.V.A.); svassiliou@chem.uoa.gr (S.V.); 2 Theoretical and Physical Chemistry Institute, National Hellenic Research Foundation, 48 Vassileos Constantinou Avenue, 11635 Athens, Greece; 3 Laboratory of Physical Chemistry, Department of Chemistry, National and Kapodistrian University of Athens, Panepistimioupolis Zografou, 11571 Athens, Greece

**Keywords:** thiosemicarbazones, thiocarbohydrazones, NMR, DFT

## Abstract

Thiosemicarbazones and thiocarbohydrazones are key sulfur-containing organic compounds known for their diverse biological, pharmaceutical, and industrial applications. Beyond their well-established therapeutic potential, their strong chelating ability allows them to form stable complexes with transition metals, enabling uses in catalysis, corrosion inhibition, and dyeing processes. Their structural characteristics and dynamic conformations critically influence both biological activity and industrial performance, making nuclear magnetic resonance (NMR) spectroscopy an indispensable tool for their analysis. This review provides a comprehensive overview of the conformational and functional properties of bioactive thiosemicarbazones and thiocarbohydrazones, with a focus on how experimental NMR techniques are used to investigate their conformational behavior. In addition to experimental findings, available computational data are discussed, offering complementary insights into their structural dynamics. The integration of experimental and theoretical approaches offers a robust framework for predicting the behavior and interactions of these compounds, thereby informing the rational design of novel derivatives with improved functionality. By highlighting key structural features and application contexts, this work addresses a critical gap in the current understanding of these promising agents across both biomedical and industrial domains.

## 1. Introduction

In our daily lives, biologically active compounds play a crucial role, particularly in medicinal and pharmaceutical sciences. Among these, nitrogen- and sulfur-containing organic compounds have attracted significant attention due to their diverse biological activities and structural versatility. Many biologically relevant molecules incorporate functional groups capable of interacting with biological targets, making them valuable for drug design and development.

**Scheme 1:**
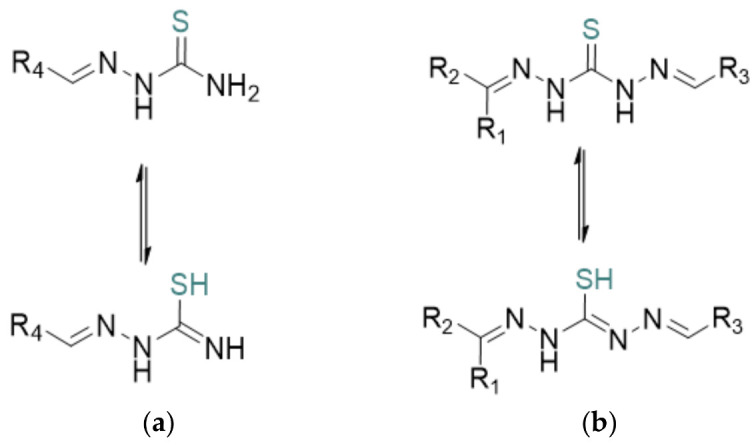
General structure of thiosemicarbazone (**a**) and thiocarbohydrazone (**b**) showing the tautomeric equilibrium between the thioketo and thioenol forms. R represents general substituent groups [6].

One important category of bioactive compounds, which has attracted considerable interest among researchers, is thiosemicarbazones (Scheme 1a) [1]. Thiosemicarbazones represent a large group of thiourea derivatives and have been evaluated for their biological abilities. The (NH_2_-CS-NH-NH_2_) group appears to be fundamental for the anticancer activity of these compounds. Thiocarbohydrazones are an important, though less studied, class of molecules possessing a set of applications like biochemical, pharmaceutical and industrial [2]. Industrially, their strong chelating ability enables the formation of metal complexes such as Ni(II)-, Cu(II)-, and Fe(III)-thiocarbohydrazones, which are used in catalysis, corrosion inhibition, and analytical chemistry. This is due to the unique balance of electronic properties, structural adaptability, and bioavailability, making them attractive candidates for drug development. Of utmost importance is their metal-chelating ability, which plays a key role in their biological and catalytic activities [3,4,5].

Thiosemicarbazones represent a class of small molecules with various pharmacological properties [7], including antiviral [8], antibacterial [9], and antitumor activities [10,11,12]. The biological properties of thiosemicarbazones have been extensively studied in pharmaceutical chemistry, particularly in inorganic medicinal chemistry, due to their ability to act as chelating agents for metal ions and the crucial role that metal coordination plays in their biochemical activity. While thiosemicarbazones exhibit wide pharmacological versatility as a general class of compounds, their activity can be further refined and directed toward specific therapeutic applications [13]. For instance, thiosemicarbazones can function as enzyme inhibitors either by complexing with endogenous metals or through redox reactions. It has been documented in the literature that they can form complexes with metal cations, leading to the creation of chelate compounds [14,15,16,17].

In addition to thiosemicarbazones, researchers have increasingly turned their attention to thiocarbohydrazones (Scheme 1). The first synthesis of these compounds dates back to 1925 [18]. Structurally, they can be classified into symmetric and asymmetric types. These compounds serve as valuable systems for ligands that exhibit a variety of biological applications [19,20,21,22,23]. They typically act as neutral or negatively charged ligands, coordinating with metals through the sulfur atom and the nitrogen atom of the double bond. This property arises from the tautomeric equilibrium between the thione (thioketo) and thiol (thioenol) forms [24,25,26,27]. Thiocarbohydrazones are also structurally related to thiosemicarbazones but incorporate a thiocarbohydrazide backbone, offering a more flexible framework with multiple potential donor sites for bonding. Both classes of compounds exhibit strong metal-chelating abilities, forming stable complexes with a variety of transition and non-transition metals. This study aims to deepen our understanding of their chemical behavior and expand the scope of their applications, particularly through the conformational analysis and characterization of thiosemicarbazone and thiocarbohydrazine derivatives.

## 2. Results and Discussion

As was mentioned before, several thiosemicarbazones and thiocarbohydrazone derivatives were found in the literature and they were studied through spectroscopic and in silico calculations. NMR and conformational analysis are powerful tools for understanding the structural dynamics of organic compounds, providing insights into tautomeric equilibria, hydrogen bonding, and molecular flexibility in solution [28,29,30,31,32]. Although in vitro studies typically refer to biological assays, theoretical calculations serve as an in silico approach to validate and support experimental findings, bridging the gap between molecular structure and reactivity. The compounds were chosen according to their favorable biological results and also due to their availability of comprehensive spectroscopic and structural data (such as IR, NMR, MS, and X-ray crystallography, where applicable). Below, the thiosemicarbazone derivatives are first presented, followed by the thiocarbohydrazones. In both sections the molecules have been organized so as to start with the simpler structures and progress to the more complex ones.

### 2.1. Thiosemicarbazones

#### 2.1.1. Structure Insights from NMR and X-Ray Crystallography

The synthesis and analysis of two novel Schiff base ligands with thiosemicarbazone structures were performed by Nehar et al. [33]. These ligands were characterized using NMR, which provided detailed insights into their structural conformation, an important factor for understanding how they interact at the molecular level.

NMR data, particularly ^1^H NMR, indicated the planarity of these ligands and their binding through hydrogen bonds, which was further confirmed uisng single-crystal X-ray diffraction. These findings suggest a stable planar structure with specific interactions, such as N-H-S- and O-H-S bonds, affecting their conformational stability and reactivity. This structure and conformational stability play a significant role in the compounds’ catecholase-like activity—an ability to catalyze the oxidation of catechols, observed in the study through the oxidation of 3,5-di-tert-butylcatechol (3,5-DTBC).

**Scheme 2:**
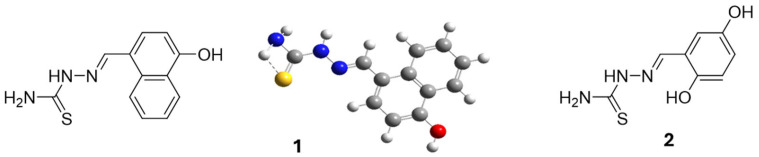
The conformation of ligand 1 (**1**) with a hydrogen bond (shown by dashed lines) between NH (blue) and S (yellow). Of importance is the linearity of the molecule, as indicated in the scheme, which contributes significantly to the drug’s activity. For **1**, a 3D presentation is given that highlights the H···S hydrogen bond, while the blue represent nitrogens, red oxygen and yellow oxygen.

The hemolytic activity of these ligands was also examined, showing that ligand L1 (Scheme 2) exhibited concentration-dependent hemolytic activity, while L2 (Scheme 2) did not show such effects. This distinction highlights how minor structural differences in these ligands can significantly affect their biological interactions and safety profiles.

The research, therefore, offers both chemical and biomedical insights, using NMR and conformational analysis to connect structural characteristics with catalytic and biological activities, making these ligands of interest for future catalytic and therapeutic applications. Specifically, for ligand 1, single-crystal X-ray analysis revealed a planar molecular structure stabilized by N-H···S and O-H···S hydrogen bonds (Scheme 2). These structural features are pivotal in understanding the ligands’ biological activities. The planarity and hydrogen bonding can influence the ligands’ ability to coordinate with metal ions, thereby affecting their catecholase-like activity. Additionally, such structural characteristics may impact interactions with biological membranes, correlating with the observed hemolytic activity, where ligand 1 exhibited concentration-dependent hemolysis, whereas ligand 2 showed no hemolytic effect [33]. Future work could focus on synthesizing derivatives that maintain this planarity to optimize both catalytic and biological performance.

Although Nehar et al. [33] reported promising catecholase and mild hemolytic activity, further structural modifications could enhance biological performance. Introducing electron-donating or -withdrawing groups on the aromatic ring may improve lipophilicity and membrane interaction. Metal complexation, particularly with Cu(II) or Zn(II), could boost activity through increased stability and reactivity. Additionally, conformational analysis via 2D NMR (e.g., NOESY, HSQC) may reveal structure–activity relationships that guide the design of more bioactive isomers. Hybridization with known pharmacophores also represents a potential route to enhanced therapeutic effects.

**Scheme 3:**
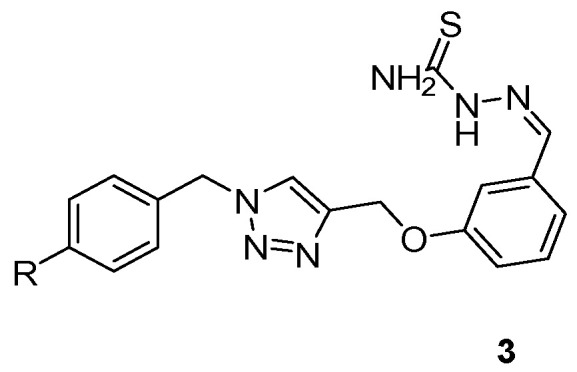
General structure of the synthesized thiosemicarbazone–triazole hybrids (**3**), where R represents various substituted phenyl groups.

Similarly, Naveen et al. [34] synthesized a series of 1,4-disubstituted-1,2,3-triazole-thiosemicarbazone (TSC) hybrid molecules targeting dual functionality and improved pharmacological profiles (Scheme 3). These hybrids demonstrated structural stability and biological promise based on their core architecture [34].

In the work of Naveen et al., these hybrids exhibited notable antimicrobial activity against Gram-positive and Gram-negative bacteria, along with significant anticancer effects against selected human cancer cell lines, underscoring their dual-function potential in drug development.

**Scheme 4:**
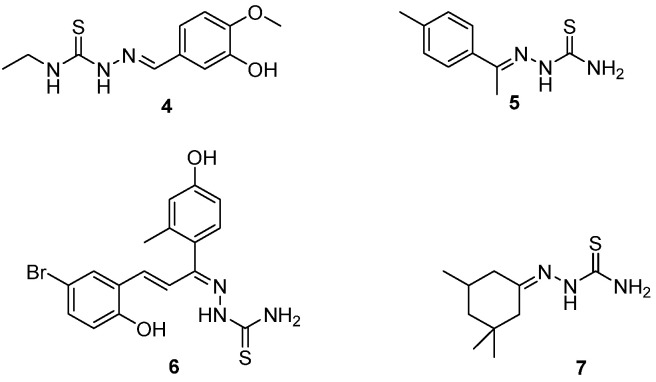
Structures of thiosemicarbazones studied by Yakan et al. [35].

In a related study by Yakan et al. [35], several thiosemicarbazone derivatives were investigated using density functional theory (DFT) calculations to analyze and optimize their molecular geometries. As shown in Scheme 4, these compounds (**4**–**7**) exhibit structural features that are relevant for understanding conformational stability and electronic behavior. The DFT-calculated data supported experimental NMR findings by providing a theoretical basis for observed chemical shifts and bond lengths. The calculations revealed energetically favored conformers that often benefited from intramolecular hydrogen bonding or minimized steric hindrance. Notably, substitution patterns on the aromatic ring, such as electron-donating methoxy or hydroxyl groups, were associated with increased electron density on the azomethine nitrogen, which may enhance metal-chelating ability. Conversely, electron-withdrawing substituents (e.g., bromo) shifted electron density toward the thiosemicarbazone moiety, potentially influencing both cytotoxic and antimicrobial activity. This conformational insight allowed for the evaluation of how small changes in substituents affect the 3D structure and, potentially, the biological activity of the molecules. Furthermore, DFT analysis shed light on the electronic distribution within these molecules, which is critical for predicting reactivity, especially in the context of pharmaceutical development [35,36,37]. Substituent effects on the aromatic ring were shown to significantly influence both the conformation and electronic properties, thereby impacting the molecule’s interaction with biological targets.

To further enhance the biological activity of the thiosemicarbazone-based hybrids, future work could focus on introducing electron-donating or withdrawing groups to improve binding affinity, forming metal complexes to boost activity and stability, and rigidifying the scaffold to lock in bioactive conformations. These modifications, guided by structure–activity relationships and docking insights, could significantly improve enzyme inhibition and therapeutic potential.

#### 2.1.2. Synthesis and Characterization of New Thiosemicarbazone Derivatives

**Scheme 5:**
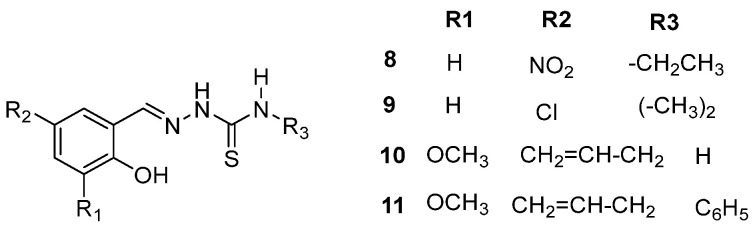
Thiosemicarbazones synthesized from Hussein et al.

Hussein et al. [38] synthesized and characterized new thiosemicarbazone derivatives (**8**–**11**), emphasizing their anti-inflammatory potential (Scheme 5). Specifically, IR and 1D NMR experiments were performed using DMSO-d_6_. In the study on thiosemicarbazone derivatives, researchers synthesized and characterized two new compounds, confirmed by various spectroscopic techniques, including ^1^H and ^13^C NMR spectroscopy. The study found that these compounds exhibit strong conformational stability and favorable electronic structures, influenced by substituents such as nitro, chloro, methoxy, allyl, and phenyl groups, enhancing their potential for COX inhibition and anti-inflammatory activity.

These compounds also demonstrated significant anti-inflammatory potential in vitro, effectively inhibiting protein denaturation, a common pathway in inflammation, and interacting with COX-2, a key enzyme in the inflammatory process. The study’s findings suggest these thiosemicarbazone derivatives hold promise for further drug development in anti-inflammatory therapies [38].

**Scheme 6:**
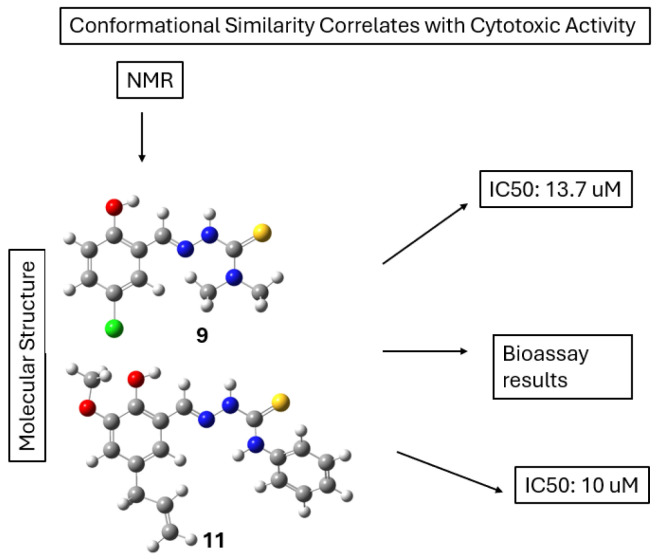
NMR analysis shows that compounds **9** and **11** adopt similar conformations in solution. These conformations correlate with their comparable cytotoxic activities, as reflected by IC50 values of 13.7 μM and 10 μM, respectively.

This structural information directly influenced the compounds’ biological activity, such as their enzyme inhibition or antimicrobial properties, by optimizing interactions with biological targets (Scheme 6).

#### 2.1.3. Structural and Biological Analysis of 2,6-Disubstituted Thiosemicarbazone Derivatives

**Scheme 7:**
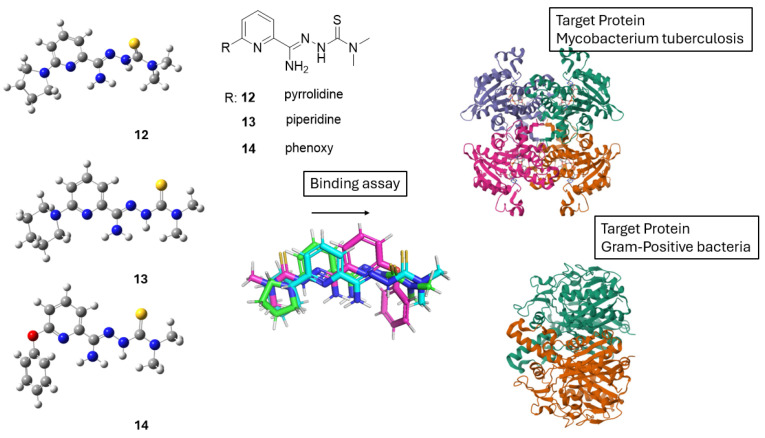
Structures of thiosemicarbazone derivatives **12**–**14** with variable R substituents studied by Ziembicka et al. [39]. Crystallographic alignment shows significant conformational overlap, indicating a conserved bioactive geometry. Docking studies reveal that this shared conformation facilitates binding to both Mycobacterium tuberculosis and Gram-positive bacterial target proteins, supporting a broad-spectrum antibacterial mechanism of action.

Ziembicka et al. [39] synthesized and analyzed three distinct 2,6-disubstituted TSC derivatives bearing pyrrolidine, piperidine, and phenoxy substituents (**12**–**14**). The study focused on three derivatives with varying substituents and examined their structural and biological properties (Scheme 7). Using NMR in tandem with X-ray crystallography, the study highlighted the zwitterionic character of the compounds in the solid state [39].

The biological activity of the 2,6-disubstituted thiosemicarbazone derivatives is closely linked to their chemical structure. Variations in the substituents (e.g., pyrrolidine, piperidine, and phenoxy groups) affect the compounds’ ability to interact with biological targets, enhancing their antimicrobial and antimycobacterial properties. These derivatives showed significant activity against *Mycobacterium tuberculosis* and Gram-positive bacteria, with low cytotoxicity toward human cells. Additionally, favorable ADME properties suggest good bioavailability, supporting their potential as therapeutic agents. Overall, structural modifications play a key role in optimizing their biological effectiveness (Scheme 7).

The biological activity of these derivatives strongly correlated with their chemical structure. Substituent variations modulated antimicrobial and antimycobacterial efficacy, showing significant activity against Mycobacterium tuberculosis and Gram-positive bacteria, alongside low cytotoxicity toward human cells. To improve the biological activity of the 2,6-disubstituted thiosemicarbazone derivatives, future studies could explore introducing functional groups that enhance membrane permeability or target-specific interactions, as well as forming metal complexes to exploit potential synergistic effects. Additionally, fine-tuning the molecular structure based on ADME profiles and crystal structure data could lead to more potent and selective bioactive compounds.

**Scheme 8:**
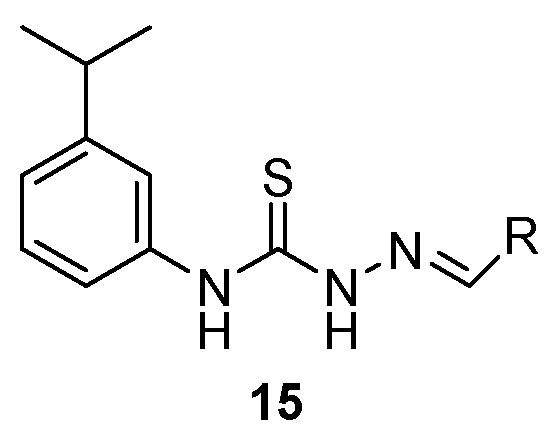
Representative thiosemicarbazone derivatives with varying aromatic substituents indicated by R. Synthesized by Qi et al. [40].

In another study by Qi et al. [40], investigated a series of thiosemicarbazone derivatives using NMR spectroscopy in DMSO-d_6_ to assign structural features in Scheme 8 [40].

**Scheme 9:**
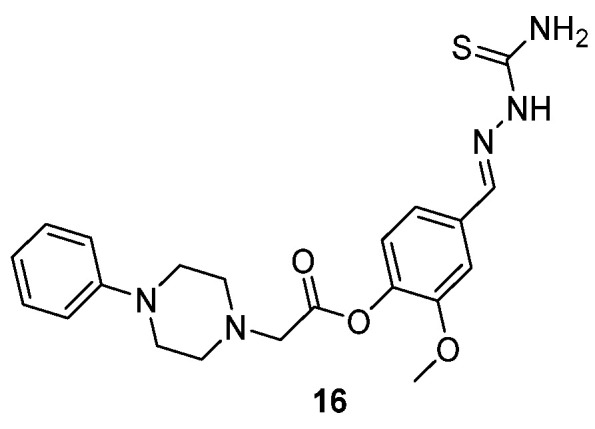
Structure of a representative thiosemicarbazone derivative synthesized from Tokali et al. (**16**) [41].

One example of the compounds that Tokali et al. [41] studied is shown in Scheme 9 (compound **16**). Furthermore, modifying the substitution pattern or introducing heterocyclic moieties may enhance binding affinity and selectivity, offering opportunities for improved pharmacological profiles.

**Scheme 10:**
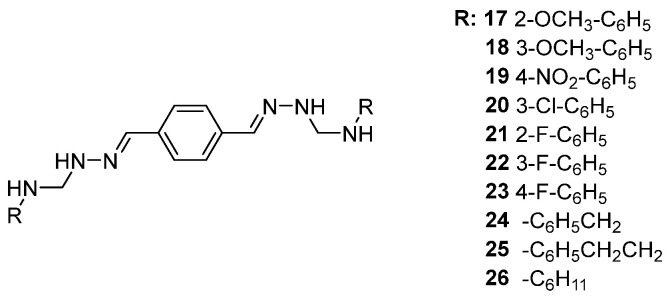
Structures of bis(semicarbazones) examined by Yakan et al. [42].

Continuing this line of investigation, Yakan et al. [42] examined the ^1^H NMR spectra of derivatives **17**–**26**, which displayed characteristic aromatic proton signals in the range of 6.5–8.0 ppm. One of them are downfield resonances between 9.0 and 11.0 ppm attributable to -NH and -OH protons involved in intramolecular hydrogen bonding (Scheme 10).

The electronic absorption spectra of the new bis(semicarbazones) derived from Schiff bases (**17**–**26**) displayed absorption bands within 256–406 nm in dimethylsulfoxide. These spectra revealed notable absorption bands corresponding to the aromatic ring (C=C) and the thiosemicarbazone (C=S)–imine (CH=N) regions, attributed to π → π* and n → π* transitions. The π → π* transitions of C=C in the aromatic ring were observed at 256 nm, except for compound 14, which showed this band at 258 nm, characteristic of the benzenoid structure.

**Scheme 11:**
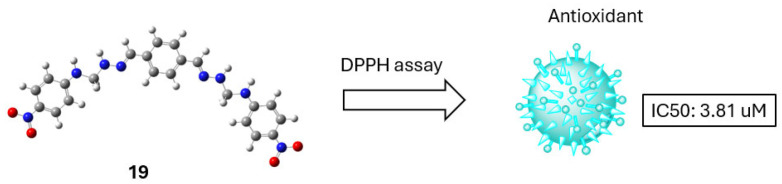
Most stable conformation of compound **19**, which demonstrated the best antioxidant activity (IC50 = 3.81 µM, DPPH assay).

The n → π* transitions of the imine (C=N) were detected at 282–296 nm across all compounds, while bands at 352–406 nm corresponded to n → π* transitions of the thiosemicarbazone moiety (C=S). In compound 21, three n → π* transitions of the thiosemicarbazone appeared at 352 nm (shoulder), 370 nm, and 390 nm. Additionally, the n → π* transition of the azomethine (C=N) was noted at 286 nm (shoulder), and the π → π* transition of the aromatic ring (C=C) appeared at 256 nm. The antioxidant activity of the synthesized compounds was evaluated using the DPPH (2,2-diphenyl-1-picrylhydrazyl) radical scavenging assay, which measures the compounds’ ability to neutralize free radicals. The results showed that all compounds exhibited antioxidant activity, though generally lower than that of the standard, ascorbic acid. The IC50 values, representing the concentration required to scavenge 50% of DPPH radicals, ranged from 3.81 ± 0.01 to 29.05 ± 0.11 μM, indicating varying degrees of potency. Among them, compound 19 demonstrated the highest antioxidant activity (Scheme 11). The 3D conformation of compound 19, shown in Scheme 11, suggests that its folded structure and extended conjugation may enhance radical scavenging ability. These findings correlate with the UV–Vis spectral data, where absorption features associated with conjugated systems and electron-donating groups may contribute to the observed radical scavenging capacity [42].

#### 2.1.4. Structural Characterization of More Complex Thiosemicarbazone Derivatives

**Scheme 12:**
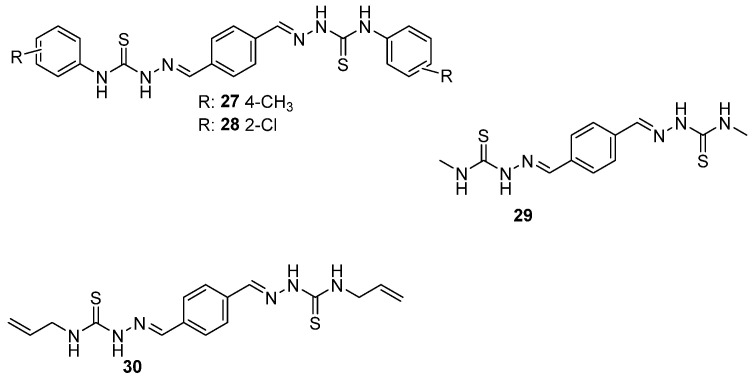
Structures of compounds **27**–**30** synthesized from Muğlu et al. [43].

Muğlu et al. [43] synthesized thiosemicarbazones derivatives (**27**–**30**), which are shown in Scheme 12. They measured the ^1^H NMR spectra, which provided insights into the molecular geometry and electronic environment of the hydrogen atoms in the compounds, particularly those involved in hydrogen bonding. Conformational analysis was further supported by density functional theory (DFT) calculations, which explored the compounds’ stability and antioxidant potential. By comparing the NMR results with DFT predictions, the study examined the conformational preferences and their influence on the compounds’ ability (Scheme 12) to act as antioxidants via Hydrogen Atom Transfer (HAT) and Single-Electron Transfer (SET) mechanisms. Τhe DFT analysis indicated that the antioxidant behavior of these compounds is concentration-dependent, with different substituents influencing the preferred mechanism (HAT or SET). The study showed that substituents at the aromatic ring, particularly electron-donating groups like –OH, increased the compound’s stability and antioxidant efficiency [43].

**Scheme 13:**
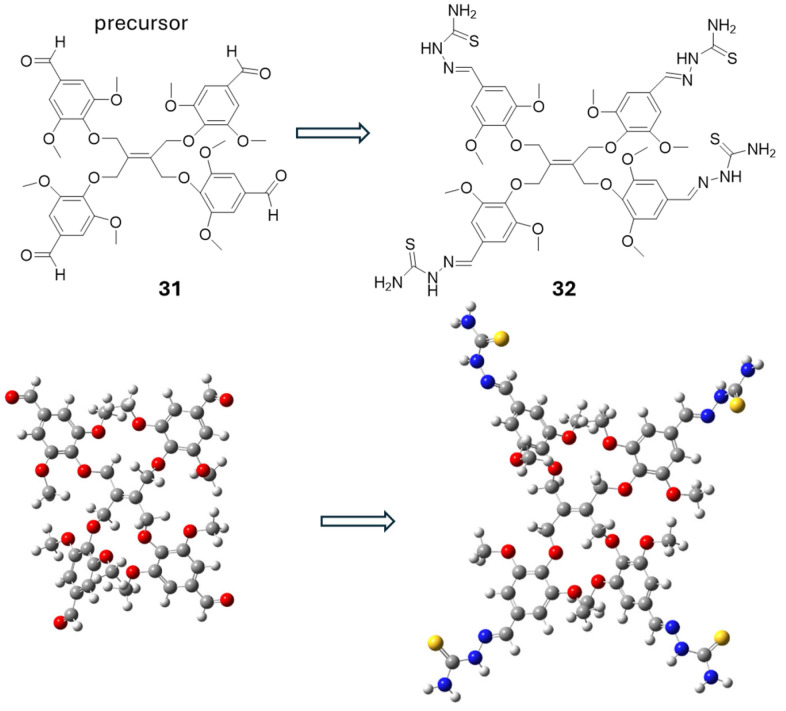
Low-energy conformers of compounds containing thiosemicarbazone and aldehyde groups [44].

Moreover, Karakurt et al. [44] synthesized thiosemicarbazone derivatives by reacting thiosemicarbazide with various aldehyde precursors (**31**–**32**). The structures are shown in Scheme 13. NMR and conformational analysis played a crucial role in confirming the structure and understanding the behavior of the synthesized compounds. ^1^H and ^13^C NMR spectra were used to identify the chemical environments of the protons and carbons within both the newly synthesized aldehyde precursors and the resulting thiosemicarbazone derivatives. DMSO-d_6_ was used as a solvent. Conformational preferences of the molecules were also investigated, which were critical for determining their biological activity (Scheme 13) [44]. Also, they explored how the nature of the aldehyde precursors influences the antioxidant behavior of the thiosemicarbazone derivatives. Compounds with electron-withdrawing aldehyde groups exhibited lower antioxidant efficiency than those with electron-donating substituents. The conformations of the thiosemicarbazone–aldehyde derivatives were determined using X-ray crystallography and supported by molecular docking studies. These conformations affect biological activity by influencing how well the molecules interact with target sites, such as enzymes. Planarity and hydrogen bonding play key roles in stabilizing active forms. To enhance activity, structural modifications like adding electron-donating/withdrawing groups, rigidifying flexible parts, or forming metal complexes can improve binding and biological effectiveness. Scheme 13 illustrates the low-energy conformers synthesized from thiosemicarbazone and aldehyde precursors. It highlights how structural modifications lead to more stable geometries, which can enhance binding affinity and biological activity.

### 2.2. Thiocarbohydrazones

#### 2.2.1. Study of Conformational Isomerism in Thiocarbohydrazone Compounds

**Scheme 14:**
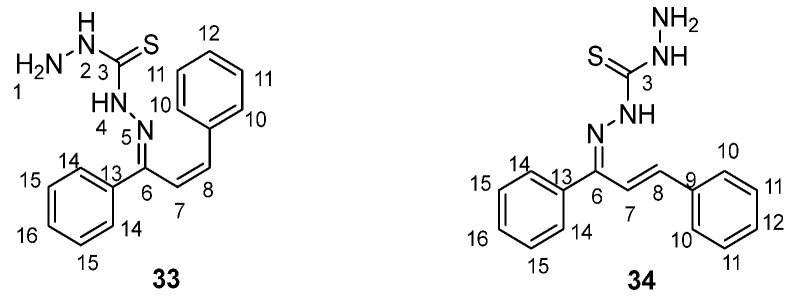
Two-dimensional low-energy conformers of KKI18 (**33** in *cis* and **34** in *trans* for the double bond **7**–**8**).

Following the study of thiosemicarbazones, thiocarbohydrazones derivatives were studied. Specifically, compound KKI18 was studied by Georgiou et al. [45]. Specifically, it adopts two low-energy conformations (Scheme 14).

**Scheme 15:**
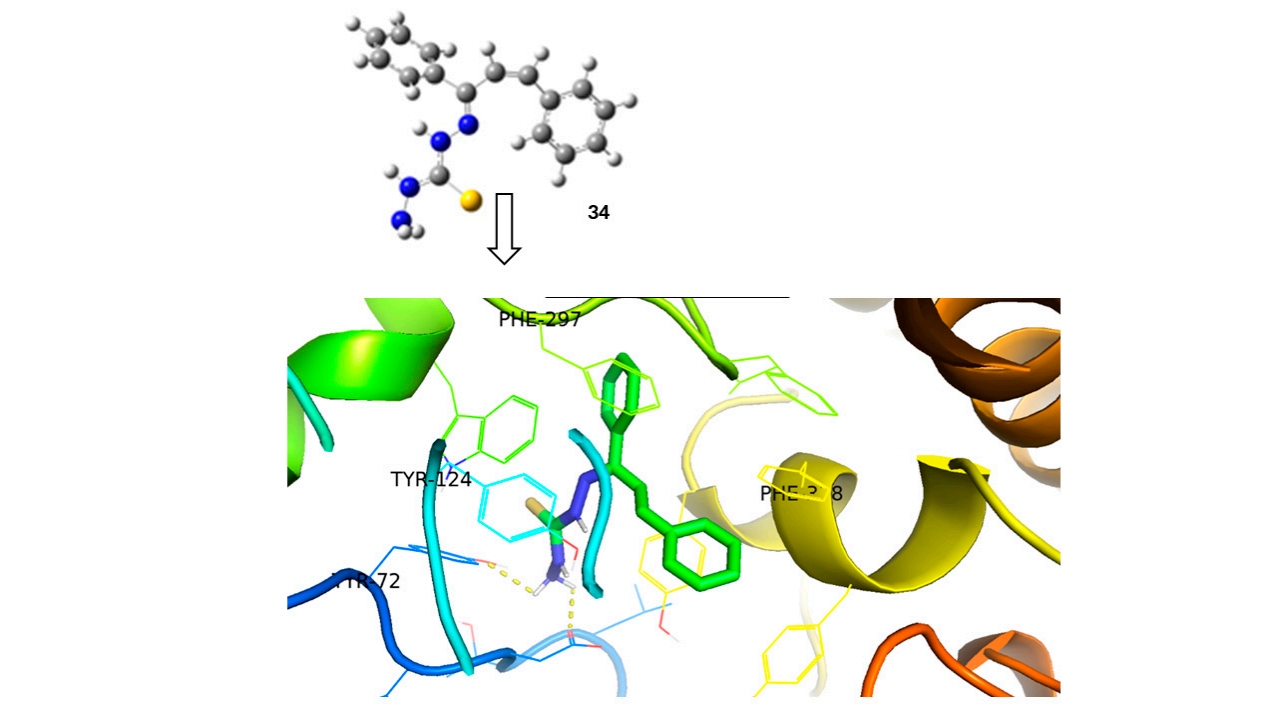
Predicted binding pose of the low-energy conformer of compound KKI18 (34) at the active site of acetylcholinesterase, an enzyme implicated in neurological disorders such as Alzheimer’s disease. The ligand (shown in ball-and-stick representation) interacts with key residues including **TYR124**, **TRP72**, **PHE297**, and **PHE338** (highlighted in stick form). Hydrogen bonds and other non-covalent interactions are represented by dashed yellow lines. Although some structural elements overlap in the 3D view, the critical interactions are: (i) hydrogen bonding with **TYR124** and **TRP72**, and (ii) π–π stacking interactions with **PHE297** and **PHE338**. The predicted binding energy (−9.05 kcal/mol) reflects a favorable ligand–receptor interaction. Binding energy refers to the free energy change upon ligand–receptor complex formation and is reported here in kcal/mol.

The conformational flexibility of this compound plays a significant role in its binding interactions with enzymes, such as acetylcholinesterase. Docking simulations revealed that the low-energy conformer exhibited favorable binding energy (−9.05 kcal/mol), with key hydrogen bonding interactions at the enzyme’s active site (Scheme 15). These conformations directly affect the molecule’s pharmacokinetic and toxicity profiles, as indicated by in silico ADMET predictions [45]. Finally, the structure assignment and conformational analysis were achieved by applying homonuclear and heteronuclear 2D nuclear magnetic resonance (NMR) spectroscopy (2D-COSY, 2D-NOESY, 2D-HSQC, and 2D-HMBC) and density functional theory (DFT) The obtained DFT lowest-energy conformers agreed with the NOE correlations observed in the 2D-NOESY spectra.

#### 2.2.2. Spectroscopic Investigation of Substituted Thiocarbohydrazones

**Scheme 16:**
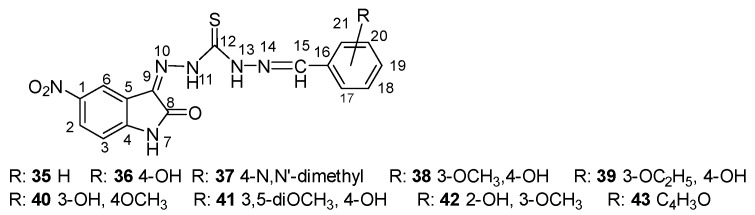
The structure of compounds **35**–**43** synthesized by Yakan et al. [46]. The imine double bond is in the *Z*-configuration.

Later studies by Yakan et al. [46] showed that thiocarbohydrazones are generally prepared from the condensation of primary amines and active carbonyl groups **35**–**43** (Scheme 16). Schiff bases are an important class of compounds in the pharmaceutical and biological fields [47,48,49,50,51,52]. Schiff bases of isatin are known to possess a wide range of pharmacological properties including antibacterial, anticonvulsant, anti-HIV and antifungal activity [53,54].

**Scheme 17:**
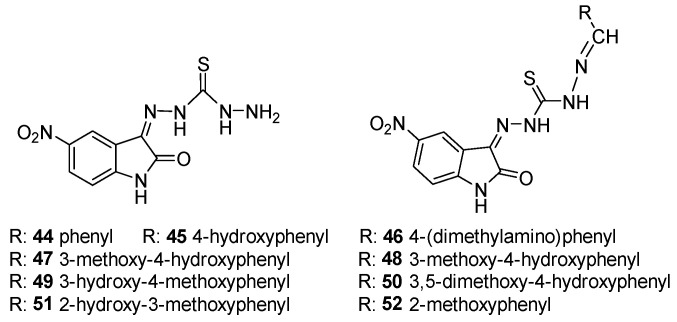
Structure of 5-nitroisatin-β-thiocarbohydrazone (**left**) and Schiff bases based on 5-nitroisatin-β-thiocarbohydrazone (**right**).

**Scheme 18:**
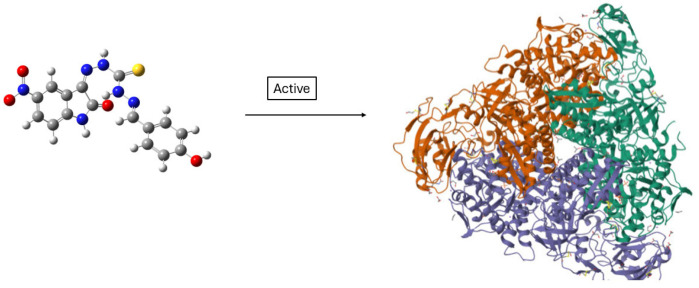
Predicted binding low-energy conformer of compound **45** in Jack Bean urease (PDB ID: 4GY7) [55]. The binding pocket is highlighted in the protein surface, and the ligand is shown in ball-and-stick representation. Compound **45** was shown to be active against this enzyme.

For compounds **44**–**52** (Scheme 17), the -C=S (C13) signal of thiocarbohydrazone moiety was observed at a range of *δ* 174.5–175.7 ppm, and the -C=O (C8) signal of the isatin region was detected at a range of *δ* 163.1–163.6 ppm. Furthermore, DFT is employed in conjunction with molecular docking studies to explore how the Schiff bases interact with target enzymes, providing a deeper understanding of their binding affinities and mechanisms of enzyme inhibition. As shown in Scheme 18, docking studies revealed that compound 45 fits well within the active site of Jack Bean urease, forming hydrogen bonds with key catalytic residues such as His519 and Ni-coordinated Asp494. These interactions likely contribute to its observed urease inhibitory activity.

**Scheme 19:**
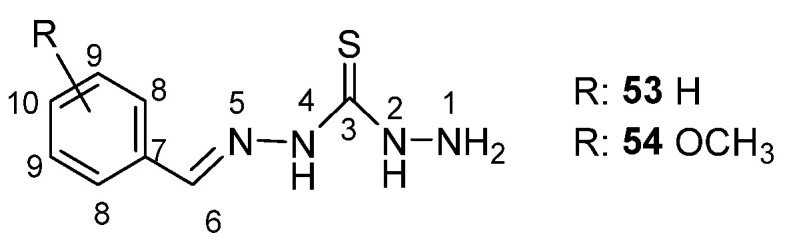
Structure of thiocarbohydrazone.

Moreover, some other thiocarbohydrazone derivatives were studied by the same group (Scheme 19). The assignment of each element (Scheme 19) is shown below [56].

**Scheme 20:**
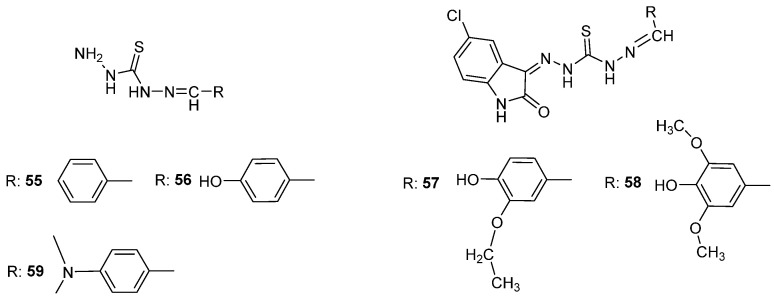
Structures of thiocarbohydrazones examined by Bakir et al. [57].

Furthermore, Bakir et al. [57] examined the FT-IR spectrum of the synthesized compounds **55**–**59** (Scheme 20). The -NH stretching vibration of the isatin ring was detected in the 3083–3187 cm^−1^ range, the -NH stretching vibrations of the thiocarbohydrazone moiety were observed in the 3595–3120 cm^−1^ range, the C=O signal of the isatin ring was observed in the 1697–1704 cm^−1^ range, the -C=N stretching vibration appeared between 1578 and 1597 cm^−1^, the -C-N stretching vibration appeared in the 1168–1221 cm^−1^ range, the C=S signal of the thiocarbohydrazone moiety was observed between 1364 and 1386 cm^−1^, and the C-Cl stretching vibration was observed in the 817–883 cm^−1^ range. Furthermore, the -CH aromatic stretching was detected in the 2983–3035 cm^−1^ range [58].

**Scheme 21:**
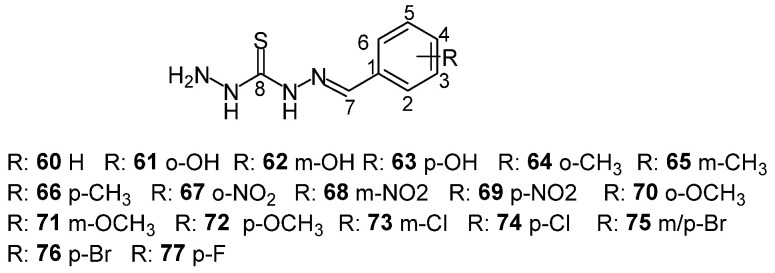
Structure of thiocarbohydrazone derivatives examined by Mrdan et al.

Thiocarbohydrazones bearing different functional groups (e.g., OCH_3_, Cl, OH) were evaluated for antioxidant and enzymatic inhibitory activity by Mrdan et al. [59] (Scheme 21). Substituent variation significantly impacted the spectral and docking results, especially regarding hydrogen bonding and π-conjugation. Compounds like **62** and **66** demonstrated well-defined conformations and favorable docking profiles. UV–Vis studies in multiple solvents showed hypsochromic shifts with increasing solvent polarity, consistent with electronic transitions influenced by solvent interactions. These results were corroborated by TD–DFT calculations, which showed strong alignment with experimental spectra. Future research should focus on improving membrane permeability, tuning binding affinity, and enhancing metabolic stability through judicious substituent selection and potential metal complexation [51,57,58,60,61].

The theoretical UV–Vis spectra were calculated on MP2/6-311G(d,p)-optimized geometries with a time-dependent (TD) density functional theory (DFT) method. TD–DFT calculations were performed in DMSO with the CAM-B3LYP functional and the 6–311G(d,p) basis set. Solvent effects were simulated using the self-consistent reaction field (SCRF)/isodensity surface-polarized continuum model (IPCM). The charge transfer distance (DCT) was estimated according to the qualitative charge transfer index method proposed by Le Bahers et al. [62].

The structure of the obtained compounds was confirmed using NMR and FT–IR spectroscopy, as well as with elemental analysis. These complementary techniques provide further validation of the functional groups and overall molecular framework of the compounds.

## 3. Conclusions

NMR spectroscopy has proven to be a powerful tool for elucidating the structural and conformational features of thiosemicarbazone and thiocarbohydrazone derivatives. Techniques such as ^1^H and ^13^C NMR effectively identify specific hydrogen and carbon environments, confirming molecular structures and offering insights into electronic effects and resonance behavior. The observed chemical shifts not only support the proposed molecular frameworks but also reveal hydrogen bonding interactions and the influence of substituents on the electronic distribution within the scaffold.

Complementary spectroscopic techniques, including IR and Raman, further support the structural assignments of newly synthesized analogs, helping to identify possible rotamers or tautomers. These shifts often point to intramolecular hydrogen bonding and electronic delocalization, both critical for understanding the stability and reactivity of these compounds.

The conformational analysis conducted for most of the studied derivatives was validated through NMR data, providing a basis for rationalizing their behavior in biological and chemical contexts. Despite these advances, further work is needed to explore the biological implications of these low-energy conformers, their structure–activity relationships, and their potential therapeutic roles. Future studies should focus on integrating spectroscopic data with computational and biological assays to unlock the full potential of this class of compounds.

**Scheme 22:**
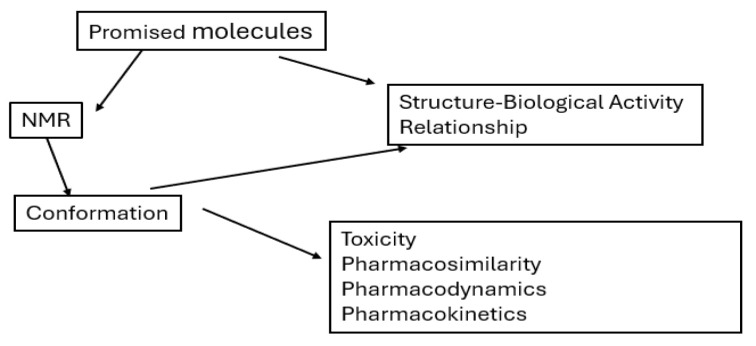
Schematic representation of the overall structure of this review, outlining the classification of thiosemicarbazones and thiocarbohydrazones, their conformational characteristics, structural diversity, and the relationship between their chemical features and biological functions.

Scheme 22 presents a conceptual diagram illustrating the relationship between promising drug molecules, their conformations, and resulting biological properties. It highlights that the conformation of a molecule, derived from its structure, plays a central role in determining its biological activity as well as key pharmacological characteristics such as toxicity, pharmacosimilarity, pharmacodynamics, and pharmacokinetics. It suggests that evaluating molecular conformation is essential for understanding how a compound will behave in a biological system, thereby supporting rational drug design and development.

Most biological data come from in silico simulating processes or in vitro studies, but translating these findings in vivo is challenging due to factors like poor bioavailability, rapid metabolism, and unpredicted toxicity. In vitro models cannot fully replicate complex biological systems, so future research should include in vivo experiments to assess pharmacokinetics, safety, and efficacy in relevant animal models before advancing to clinical trials. ADME calculations showed that the compounds are safe and bioactive.

## Data Availability

Data are provided in the paper.
